# Metabolic syndrome and Chronic Obstructive Pulmonary Disease (COPD): The interplay among smoking, insulin resistance and vitamin D

**DOI:** 10.1371/journal.pone.0186708

**Published:** 2017-10-24

**Authors:** Giuseppina Piazzolla, Anna Castrovilli, Vito Liotino, Maria Rosaria Vulpi, Margherita Fanelli, Antonio Mazzocca, Mafalda Candigliota, Elsa Berardi, Onofrio Resta, Carlo Sabbà, Cosimo Tortorella

**Affiliations:** 1 Interdisciplinary Department of Medicine, University of Bari School of Medicine, Bari, Italy; 2 Department of Cardiac, Thoracic and Vascular Science, University of Bari School of Medicine, Bari, Italy; National and Kapodistrian University of Athens, GREECE

## Abstract

**Background:**

A close relationship between Metabolic Syndrome (MetS) and Chronic Obstructive Pulmonary Disease (COPD) has been described, but the exact nature of this link remains unclear. Current epidemiological data refer exclusively to the MetS prevalence among patients with COPD and data about the prevalence of COPD in MetS patients are still unavailable.

**Aim of the study:**

To analyse and compare risk factors, clinical and metabolic characteristics, as well as the main respiratory function parameters, among patients affected by MetS, COPD or both diseases.

**Patients:**

We recruited 59 outpatients with MetS and 76 outpatients with COPD. After medical history collection, physical examination, blood sampling for routine analysis, spirometric evaluation, they were subdivided into MetS (n = 46), MetS+COPD (n = 60), COPD (n = 29).

**Results:**

A MetS diagnosis was assigned to 62% of COPD patients recruited in the COPD Outpatients Clinic of the Pneumology Department, while the COPD prevalence in MetS patients enrolled in the Internal Medicine Metabolic Disorders Outpatients Clinic was 22%. More than 60% of subjects enrolled in each Department were unaware that they suffered from an additional disease. MetS+COPD patients exhibited significantly higher C-peptide levels. We also found a positive relation between C-peptide and pack-years in all subjects and a negative correlation between C-peptide and vitamin D only in current smokers. Finally, a negative association emerged between smoking and vitamin D.

**Conclusions:**

We have estimated, for the first time, the COPD prevalence in MetS and suggest a potential role of smoking in inducing insulin resistance. Moreover, a direct effect of smoking on vitamin D levels is proposed as a novel mechanism, which may account for both insulin resistance and COPD development.

## Introduction

The Metabolic Syndrome (MetS) and Chronic Obstructive Pulmonary disease (COPD) are currently widespread clinical conditions with a significant impact on public health. The incidence of both disorders will likely increase in the next future, imposing a growing burden on the global economy. A significant association between the two disorders has been described and both epidemiological and clinical data support an important link between MetS and lung function impairment [1–3]. However, the exact nature of this relationship remains unclear. MetS is a cluster of interrelated metabolic disorders, including insulin resistance, central obesity, dyslipidemia, endothelial dysfunction, hypertension, hypercoagulation and chronic stress, that predisposes to type-2 diabetes and cardiovascular diseases (CVD) and is associated with a substantial increase in all-cause mortality [4]. It is undoubtedly a common condition, present in about 20–30% of the world’s adult population [5], whose global prevalence is rising also due to an increase in the incidence of obesity and sedentary lifestyles [3]. Scientific organizations have listed diagnostic criteria for the definition of the syndrome, with different specific cut-off points of risk values for waist circumference, blood pressure and serum levels of glucose, triglycerides and High Density Lipoprotein (HDL) [6]. Regardless of which cluster of criteria is adopted, the primary concern is to make a better identification of high-risk patients, early detection of potential cardiovascular complications and so intervene earlier. The complex interaction of genetic and environmental factors is a common feature of MetS and COPD and it seems to contribute to the onset and persistence of a low-grade systemic inflammation in both clinical conditions [7–9]. COPD is primarily characterized by persistent airflow obstruction and pulmonary inflammation, but extrapulmonary manifestations and associated comorbidities are very frequent and contribute significantly to the overall severity of the disease [10–12]. Type 2 diabetes and MetS are among the most frequently described comorbidities of COPD. Of note, insulin resistance has been involved in both pulmonary function decline and cardio-vascular complications of COPD. In addition, the association of COPD and insulin resistance has been indicated as a powerful predictor of cardiovascular mortality [11]. The prevalence of MetS in COPD has recently been systematically reviewed and the risk profile for MetS in these patients has also been outlined [13]. In several reviews of current literature, MetS was shown to be more common in younger, obese female subjects and in earlier stages of COPD, suggesting that individuals with metabolic disorders might constitute a specific COPD phenotype [13, 14], in line with the high cardiovascular-related mortality reported in mild to moderate COPD [11]. On the contrary, there is no evidence in the literature on the prevalence of COPD among patients with MetS. Although cigarette smoking does not seem to be a discriminating factor between patients with and without MetS, data from epidemiological studies on this topic are still largely conflicting. The association of tobacco use with insulin resistance and the onset of MetS is not significant in all available studies [15], while a protective effect of smoking against MetS and diabetes in women has even been described [16]. Likewise, the influence of cigarette smoke on body weight remains an unresolved issue since, in different studies, lower [17] as well as higher [18] Body Mass Index (BMI) values have been reported in current smokers.

The aim of this study was to analyze and compare clinical and metabolic characteristics, as well as the main respiratory function parameters, among patients affected by MetS, COPD or both diseases. At the same time, potential risk factors and/or pathogenic mechanisms underlying these clinical conditions were explored.

## Patients and methods

### Study population

Data were collected in 76 consecutive COPD patients attending the COPD Outpatients Clinic of the Pneumology Department, and 59 consecutive patients with MetS attending the Metabolic Disorders Outpatients Clinic of the Department of Internal Medicine, at the University of Bari Medical Center. Exclusion criteria were any kind of cancer within less than five years prior to the study, infections (including respiratory tract infections or exacerbations) and systemic corticosteroid treatment within four weeks prior to the study. All patients underwent a general examination, including the following measures: height, weight, BMI, waist circumference, arterial pressure; venous sampling for routine analysis including serum vitamin D, C-peptide and insulin assays; and instrumental tests including spirometry. Blood analysis were carried out in the same laboratory. All functional respiratory tests were performed in the Pneumology Department, whereas cardio-metabolic evaluations and measurements were carried out in the Internal Medicine Department.

COPD was diagnosed with spirometry, according to the GOLD guidelines [19]. MetS was diagnosed according to the “harmonizing definition” of the syndrome [6]. Therefore, MetS was diagnosed when at least three of the following criteria were present: 1) waist circumference ≥ 94 cm in European men or ≥ 80 cm in European women; 2) fasting glucose > 100 mg/dl or ongoing therapy for elevated glucose levels; 3) triglycerides ≥ 150 mg/dl or specific treatment for this lipid abnormality; 4) HDL < 40 mg/dl in men or < 50 mg/dl in women or specific treatment for this lipid abnormality; 5) systolic blood pressure ≥ 130 mmHg and/or diastolic blood pressure ≥ 85 mmHg or ongoing therapy for hypertension. Cardiovascular Risk (CVR) was calculated according to the European Guidelines on CVD Prevention in Clinical Practice 2012 [20]. The HOMA Index was calculated as (fasting insulin x fasting glucose)/405. The study was approved by the Clinical Investigation Ethics Committee of the University of Bari Medical Center (Ethical approval number: MSC/PBMC/2015), and all patients gave written informed consent to take part.

### Statistical analysis

Continuous variables were presented as mean ± SD; categorical variables were expressed as frequency and percentage. Evaluations of group differences were made by 1wayANOVA and chi-square test for continuous and categorical variables, respectively. Least Significant Difference Post Hoc tests were performed when necessary. Analysis of Covariance model (ANCOVA) was built to evaluate C-peptide mean levels among groups adjusted per pack-years (as covariate). ANCOVA was chosen because of two reasons: i) a linear relation between C-peptide and pack-years was hypothesized and ii) the pack-years values were different among groups. When these conditions occur covariance adjustments are suggested. The same analysis was performed for vitamin D. Linear regression was performed to evaluate C-peptide and vitamin D according to pack-years. Any association between C-peptide and vitamin D was evaluated by Pearson correlation coefficient.

Statistical analysis was performed with SPSS version 23.

## Results

**Fig 1:**
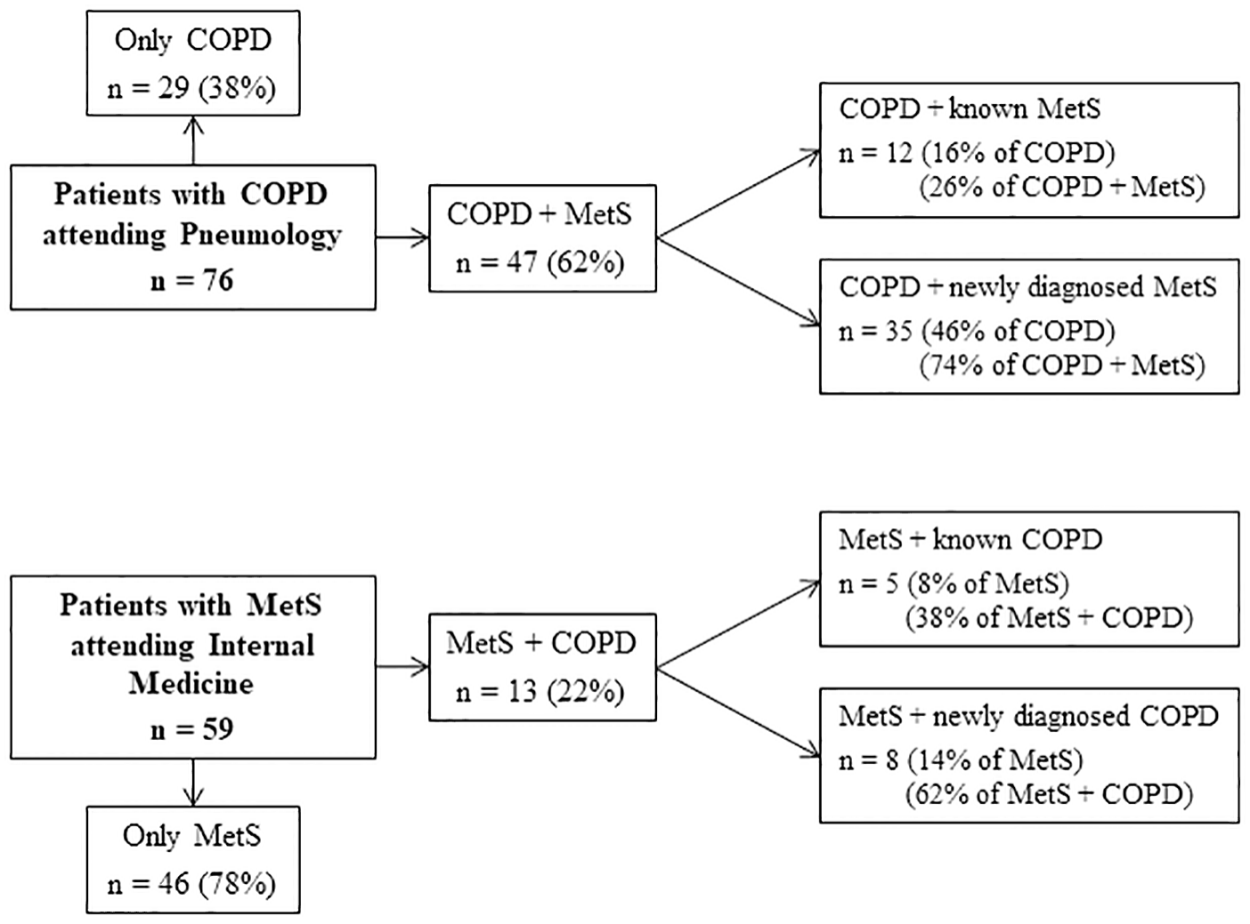
MetS and COPD prevalence in outpatients attending the Pneumology or Internal Medicine Departments.

A diagram summarizing the prevalence of MetS in COPD patients and *vice versa* is shown in Fig 1. No less than 47 of 76 (62%) COPD outpatients attending the Department of Pneumology also met the criteria for MetS. Among these, 35 (74%) were unaware that they suffered from MetS, and were diagnosed during this study. Meanwhile, the prevalence of COPD among outpatients with MetS attending the Department of Internal Medicine was 22% (13 of the 59 patients enrolled in the study). COPD was diagnosed in 62% of these patients just as a result of this investigation, so only 38% already knew that they suffered from both diseases.

Compared to COPD or MetS+COPD, patients with only MetS were younger and less likely to be smokers (Table 1). Interestingly, whereas patients with MetS, with or without COPD, showed, as expected, a higher BMI and waist circumference, more elevated systolic and diastolic blood pressure values, higher serum glucose, insulin and triglyceride levels, lower HDL and a higher HOMA Index score as compared with COPD patients, those with both MetS and COPD had significantly higher serum levels of C-peptide than those in patients with either disease (Table 1). The Cardiovascular Risk (CVR) was also higher, although not significantly, in patients with MetS+COPD. Finally, a higher prevalence of patients with GOLD stage A was found in MetS+COPD as compared with the COPD group (χ2 = 4.710, p = 0.03).

**Table 1:** Baseline characteristics of the total study population.

	MetS (n = 46)	MetS + COPD (n = 60)	COPD (n = 29)	Statistical analyses	Significance (p)
Age (years)	58.0 ± 9.5^§^	67.4 ± 8.7	68.3 ± 9.4	F = 16.64	**<0.001**
Gender, male [n (%)]	31 (67.4)	51 (85.0)	24 (82.8)	χ2 = 5.18	0.075
BMI (kg/m^2^)	31.0 ± 4.5*	30.8 ± 5.2*	25.6 ± 3.8	F = 14.45	**<0.001**
Waist circumference (cm)	107.9 ± 11.9*	105.4 ± 13.4*	90.0 ± 10.8	F = 20.04	**<0.001**
SBP (mmHg)	129.1 ± 13.8^#^	131.5 ± 10.8^¥^	123.1 ± 10.2	F = 4.65	**0.006**
DBP (mmHg)	81.0 ± 7.6^#^	81.4 ± 6.0^#^	75.2 ± 9.6	F = 6.20	**0.003**
Cardiovascular Risk (CVR) (%)	7.9 ± 6.2	12.0 ± 6.6	10.1 ± 8.9	F = 2.05	0.137
Fasting glucose (mg/dl)	115.7 ± 43.7*	112.2 ± 31.1*	89.6 ± 9.1	F = 6.13	**0.003**
HbA1c (mmol/mol)	44.5 ± 14.6	47.1 ± 14.2	38.3 ± 4.2	F = 2.74	0.07
C-peptide (ng/ml)	2.1 ± 1.0	2.8 ± 1.5^$^	2.1 ± 0.9	F = 5.58	**0.005**
Insulin (mIU/L)	13.0 ± 7.6^#^	14.5 ± 9.3^¥^	8.5 ± 4.9	F = 5.09	**0.008**
HOMA Index	3.6 ± 2.3^¥^	3.9 ± 2.4*	2.1 ± 1.3	F = 6.17	**0.002**
LDL-cholesterol (mg/dl)	100.8 ± 34.0	92.3 ± 34.7	105.7 ± 30.2	F = 1.76	0.177
HDL-cholesterol-male (mg/dl)	40.9 ± 9.8*	44.2 ± 12.6*	54.9 ± 16.2	F = 8.72	**<0.001**
HDL-cholesterol-female (mg/dl)	52.1 ± 9.4^#^	53.4 ± 15.7^#^	66.4 ± 6.8	F = 3.15	**0.048**
Triglycerides (mg/dl)	140.8 ± 72.0^#^	135.4 ± 63.6^#^	103.0 ± 42.1	F = 3.58	**0.031**
Vitamin D (ng/ml)	17.4 ± 8.1	16.0 ± 9.0	18.0 ± 7.9	F = 0.62	0.541
C-Reactive protein (mg/L)	4.1 ± 7.4	7.1 ± 17.0	4.4 ± 8.5	F = 0.83	0.439
Never smokers [n (%)]	18 (39.1)	9 (15.0)	3 (10.3)	χ^2^ = 13.08	**0.011**
Former smokers [n (%)]	14 (30.4)	29 (48.3)	17 (58.6)		
Current smokers [n (%)]	14 (30.5)	22 (36.7)	9 (31.1)		
Pack-years (n)	21.0 ± 29.6^&^	41.7 ± 33.6	38.2 ± 27.2	F = 6.17	**0.003**
FEV-1 (%pred)	102.8 ± 15.8^§^	68.8 ± 19.9	60.1 ± 24.3	F = 51.30	**<0.001**
FVC (%pred)	103.3 ± 14.6^§^	88.5 ± 21.9	85.5 ± 24.5	F = 8.58	**<0.001**
FEV-1/FVC	82.6 ± 7.7^§^	59.8 ± 10.4	52.9 ± 10.7	F = 99.08	**<0.001**
GOLD stage [n (%)]					
A		33 (57.9)	10 (35.7)	χ^2^ = 5.49	0.139
B		13 (22.8)	9 (32.1)		
C		2 (3.5)	3 (10.7)		
D		9 (15.8)	6 (21.5)		

Data are presented as mean ± SD or as frequency and percentage; Evaluation of group differences was done using 1wayANOVA and the chi-square test for continuous and categorical variables, respectively

BMI: Body Mass Index; SBP: systolic blood pressure; DBP: diastolic blood pressure; HbA1c: glycated haemoglobin; LDL: Low Density Lipoprotein; HDL: High Density Lipoprotein; Pack-years defined as twenty cigarettes smoked every day for one year; FEV-1: Forced Expiratory Volume in the first second; FVC: Forced vital capacity; GOLD: Global Initiative for Chronic Obstructive Lung Disease (2017)

Post hoc analyses were performed using Least Significant Difference

Significance vs COPD and vs MetS+COPD:

^&^p<0.05;

^§^p<0.001

Significance vs COPD:

^#^p<0.05;

^¥^p<0.01;

*p<0.001

Significance vs MetS and vs COPD:

^$^p<0.05

**Fig 2:**
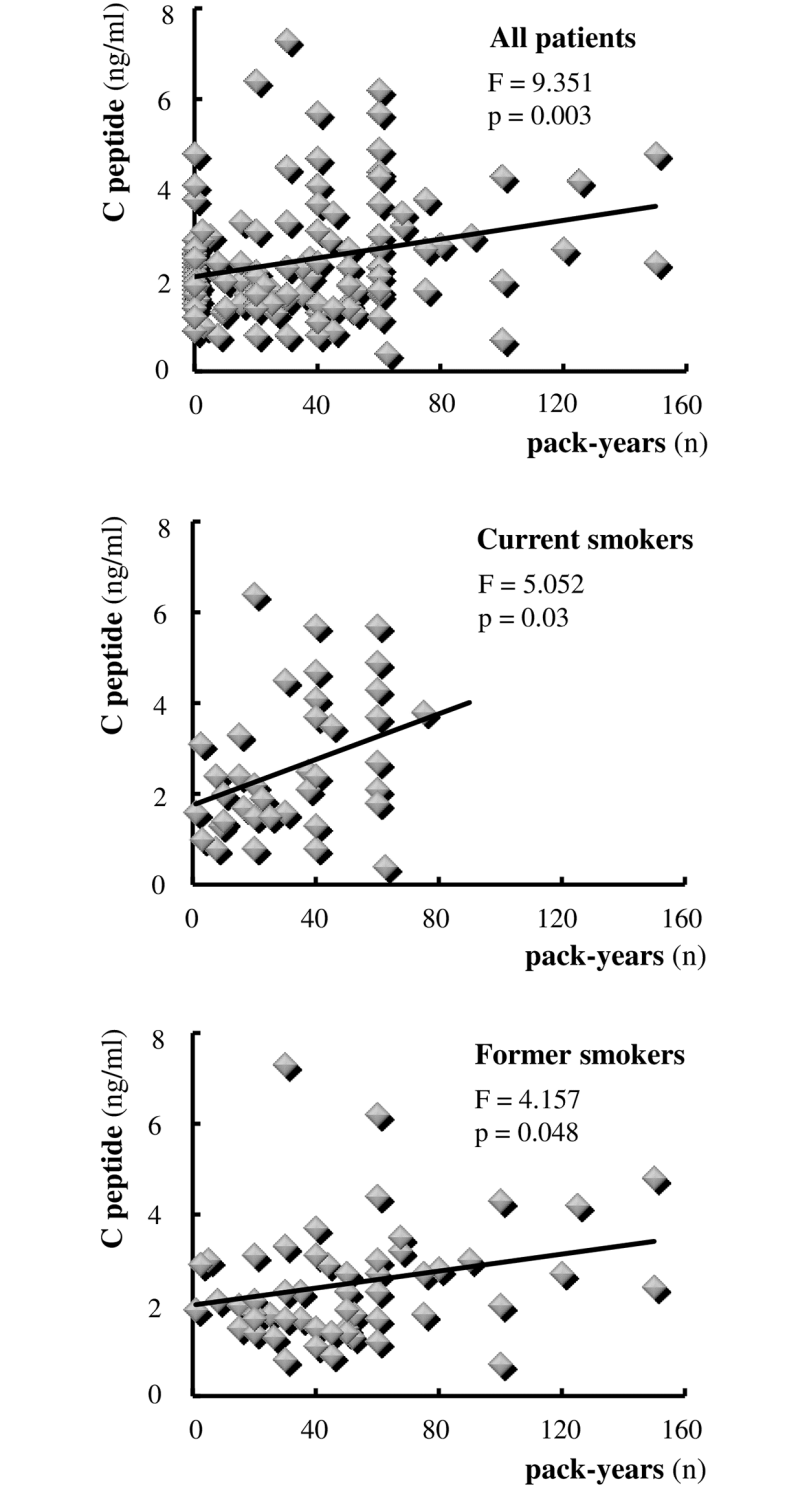
Effect of smoking on serum levels of C-peptide. Smoking is expressed in terms of number (n) of pack-years. Each symbol identifies a single individual.

Analysing these data at a glance, we were particularly impressed by the synergistic effect between MetS and COPD on serum C-peptide levels. Smoking is a major risk factor for COPD and has been related to insulin resistance, even if not conclusively. Moreover, a high percentage of our patients were former or current smokers. On these bases, we evaluated the possible effect of smoking on the levels of C-peptide. As illustrated in Fig 2, a significant positive relation between pack-years and serum C-peptide levels was observed in patients overall (model: C-peptide = 2.08 + 0.01pack-years; F = 9.351, p = 0.003). This relationship appeared particularly evident in current smokers (model: C-peptide = 1.754 + 0.025pack-years; F = 5.052, p = 0.03) but, interestingly, remained significant in former smokers as well (model: C-peptide = 2.002 + 0.09pack-years; F = 4.157, p = 0.048) (see Fig 2). Similar results were obtained when serum insulin levels or HOMA index, instead of C peptide, were related to smoking (data not shown). Multivariate analysis confirmed the difference in C-peptide circulating level means among groups adjusted per smoking habit. In fact, the effect of smoking on C-peptide levels occurred regardless of which group of patients was analyzed (ANCOVA results: group effect F = 3.922, p = 0.02; pack-years effect F = 7.473, p = 0.007; interaction effect F = 0.807, p = 0.449).

**Fig 3:**
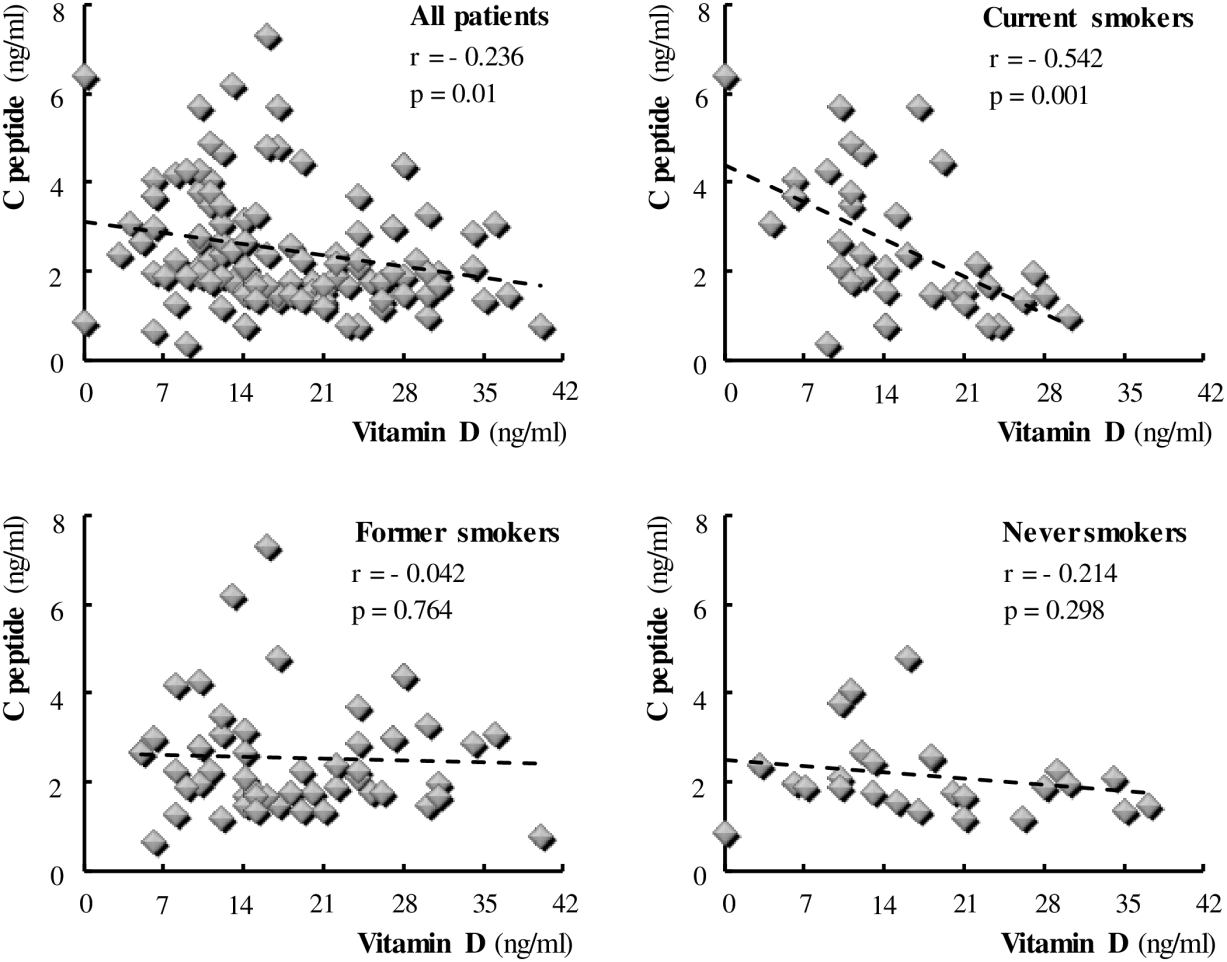
Correlation between serum levels of Vitamin D and C-peptide. Each symbol identifies a single individual.

Serum vitamin D levels were not different among MetS, COPD and MetS+COPD groups, but values were low or in the lower range of normal in most patients. This prompted us to evaluate the behaviour of vitamin D according to changing the C-peptide and/or smoking variable. The results are depicted in Fig 3, demonstrating a negative correlation between serum vitamin D levels and C-peptide (r = -0.236, p = 0.01). Detailed analysis revealed that this finding was supported only in current smokers (r = -0.542, p = 0.001), as the negative vitamin D/C-peptide correlation disappeared in former and non smokers.

**Fig 4:**
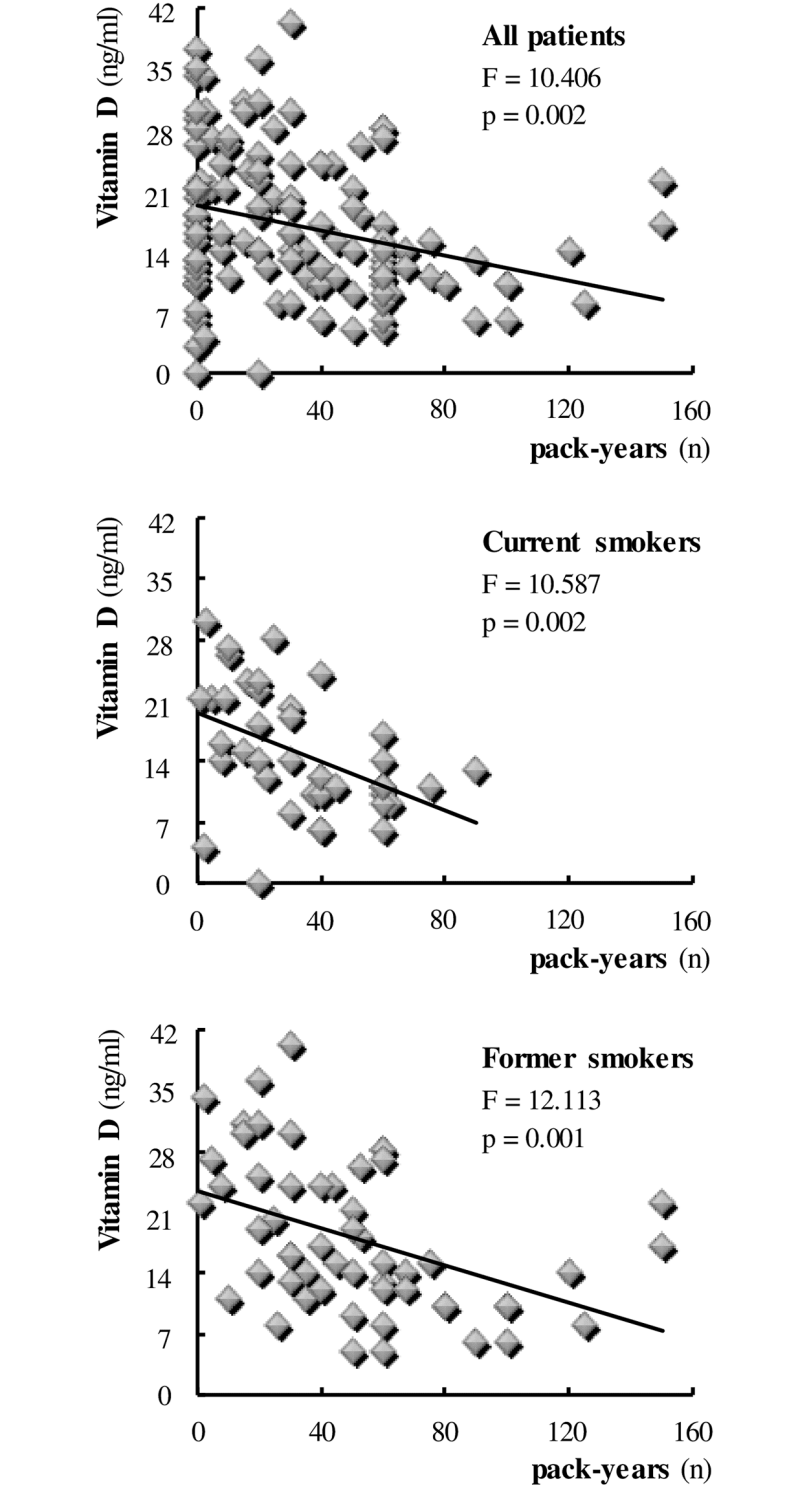
Effect of smoking on serum levels of Vitamin D. Smoking is expressed in terms of number (n) of pack-years. Each symbol identifies a single individual.

Finally, assessing the direct effect of smoking on vitamin D we found a negative relationship between the two variables in the patients overall (model: vitamin D = 19.342–0.073pack-years; F = 10.406, p = 0.002) (Fig 4). In particular, the effect of smoking in reducing vitamin D levels was equally significant in current smokers (model: vitamin D = 19.630–0.142pack-years; F = 10.587, p = 0.002) and those who had quit smoking (model: vitamin D = 23.462–0.107pack-years; F = 12.113, p = 0.001) (see Fig 4). Multivariate analysis showed no difference among groups in vitamin D level means adjusted per smoking habit, and emphasizes the effect of smoking on vitamin D levels, regardless of which group of patients was analyzed (ANCOVA results: group effect F = 0.701, p = 0.498; pack-years effect F = 9.394, p = 0.003; interaction effect F = 0.292, p = 0.747).

## Discussion

The present study confirms the significant association between the MetS and COPD and highlights new features indicating an unexpected interplay among smoking, insulin sensitivity, vitamin D and COPD. Contrary to a prevalence of MetS ranging around 25–27% in the adult Italian population, a very high percentage of COPD patients (62%) included in the study satisfied the criteria for MetS according to the harmonizing definition by Alberti *et al*. [6]. This prevalence is much higher than that observed in studies adopting more restrictive criteria for diagnosing the syndrome, but is almost in line with more recent studies based on IDF waist circumference cut-off points [3, 13]. Interestingly, available epidemiological data refer exclusively to the MetS prevalence among patients with COPD but no data are yet reported in the literature about the prevalence of COPD in subjects with a MetS phenotype. Of note, 22% of our MetS patients also had diagnosed COPD, a percentage definitely elevated when considering that the COPD prevalence in the age-matched Italian population is not higher than 8–9% in men and 4–5% in women, with a mean value of 6% in the general population [21]. This clinical aspect appears to be in line with recent experimental data showing that high insulin levels adversely affect lung structure and function [22]. Although the MetS is known to be one of the main comorbidities complicating the COPD clinical course, the surprising aspect of our data lies in the fact that among patients suffering from both pathologies, more than half received a new diagnosis of MetS (74% of patients admitted for pulmonary disease) or of COPD (62% of the MetS group) only as an effect of study enrolment. Undoubtedly, this strongly indicates the need to make a proper assessment of the metabolic status in COPD patients, as also suggested very recently by Lonardo *et al*. [23]. Likewise, a thorough evaluation of patients with the MetS should include spirometric assessment of respiratory function, even in the absence of evident symptoms. This approach would undoubtedly offer benefits from the clinical and prognostic points of view. In fact, in our patients the simultaneous presence of the two disorders posed a greater cardiovascular risk and defined a clinical phenotype characterized by an earlier stage of pulmonary disease, both conditions that benefit from timely treatment.

The detection of significantly higher levels of C-peptide in MetS+COPD patients suggested a synergistic effect exerted by the two pathologies in inducing and/or strengthening a condition of insulin resistance, known to be one of the major predictors of cardiovascular disease. A likely explanation could be the close relationship between cigarette smoking, the leading etiological agent of COPD, and insulin sensitivity, as evidenced by the rise in C-peptide levels with the increase in pack-years in current as well as former smokers. In this context, it would be worth stressing that an association has been found in active smokers with a greater insulin resistance, accumulation of visceral fat and a 26% increased risk of developing the MetS as compared with non smokers [15, 24–26], and that the risk of MetS has been reported to persist up to 20 years after quitting smoking [27]. The possible involvement of pro-inflammatory soluble mediators in modulating the link between cigarette smoking and insulin resistance is now under investigation by our group.

In recent years, vitamin D has been gaining interest in view of its many previously unsuspected biological effects beyond simply regulating the body’s mineral metabolism, including a potential role in cardiometabolic risk protection [28]. In particular, vitamin D deficiency is now recognized as a worldwide concern, and an increasing number of epidemiological studies show an inverse association between circulating vitamin D levels and cardiovascular [29, 30], oncologic and inflammatory diseases [31]. However, the underlying mechanisms are still under study and trial evidence does not conclusively support a causal role of vitamin D deficiency in such disorders. We found similar mean vitamin D concentrations in patients with the MetS, COPD or both diseases. Nevertheless, in all groups vitamin D levels appeared to be lower than normal, suggesting a possible relationship between hypovitaminosis D and both diseases. At first, the negative correlation between the levels of vitamin D and C-peptide, highlighted in all subjects, seemed to support the previously reported association of vitamin D deficiency with both insulin resistance and an increased risk of developing the metabolic syndrome [32] and type 2 diabetes [33, 34]. Surprisingly, the influence of current cigarette smoking was found to be decisive, since no significant correlation between the two variables could be confirmed in non-smokers or even in former smokers. This, in our opinion, makes a direct pathogenic role of vitamin D deficiency in insulin resistance unlikely, suggesting that the deficit acts rather by strengthening and/or mediating the effect of smoking. To shed light on the latter hypothesis, it seemed appropriate to assess the relationship between pack-years and vitamin D. In line with our assumptions, a significant effect of cigarette smoking in reducing vitamin D levels was observed in all the study patients, regardless of the baseline disease. Similarly to the above described pack-years/C-peptide relationship, in this case, too, the effect was significant not only in current but also in former smokers.

Overall, these data appear particularly intriguing and innovative also because, to our knowledge, such a picture indicating a close link between cigarette smoking, insulin resistance, vitamin D and COPD has never before been depicted in the literature. It must be said that in recent years a peculiar link between vitamin D and lung function has been increasingly highlighted. In this regard, vitamin D deficiency has been described to amplify the harmful effect of smoking on pulmonary function [35]. Furthermore, it has been associated with both a worse lung function and a faster lung function decline in smokers [35, 36] as well as in the general population [37]. Finally, in prospective analyses, low vitamin D concentrations appear to be related with a higher risk of future development of spirometrically defined COPD [37]. Therefore, Vitamin D sufficiency has been suggested to have a potential protective effect on lung function and on the rate of its decline, probably due to the anti-inflammatory and anti-oxidant properties of this versatile vitamin [38] that has also been shown, *in vitro*, to improve antibacterial defense in cigarette smoke-exposed airways [39]. Interestingly, a very recent systematic review leads to the conclusion that vitamin D supplementation protects subjects with more serious vitamin deficiency at baseline against acute respiratory tract infections, providing benefit to subjects with higher baseline concentrations as well [40]. This, in line with the newly identified mitochondria effects of vitamin D [41, 42] and recent better understanding of the role of mitochondrial dysfunction in the genesis of respiratory diseases [43], supports novel mitochondria-targeted therapeutic strategies in patients with lung disease.

A limitation of the study is represented by the small number of patients, even if the statistical significance obtained was adequate to address the main research questions. Here, we estimate, for the first time, the prevalence of COPD in patients with MetS and highlight the prognostic value of systematically assessing the coexistence of both pathologies. In addition, the evidence of a significant modulation of insulin sensitivity by smoking contributes to clarify the unresolved issue as to whether smoking may favor the development of the MetS, providing a possible explanation for the synergistic effect on metabolic status and CVR exerted by the simultaneous presence of MetS and COPD. Finally, a direct effect of smoking on vitamin D concentrations is postulated as a novel mechanism that may account for both insulin resistance and the development of COPD.

## Supporting information

S1 Dataset Minimal data set. (XLS)
